# Expert consensus on the management of third molar health

**DOI:** 10.1038/s41368-025-00413-4

**Published:** 2026-04-15

**Authors:** Rui Sun, Yifan Xu, Yang Wu, Jian Pan, Chenchen Zhou, Duohong Zou, Yujiang Wang, Yang Xue, Yu Cai, Nianhui Cui, Kaijin Hu, Wei Zhang, Bing Han, Qing Zhou, Songling Chen, Haikuo Tang, Liao Wang, Xing Wang, Bo Li, Zhigui Ma, Xiangliang Xu, Zhige Li, Ye Wu, Guowen Sun, Fudong Zhu, Yanping Hu, Kang Gao, Jian Zhou, Jihong Zhao

**Affiliations:** 1 State Key Laboratory of Oral & Maxillofacial Reconstruction and Regeneration, Key Laboratory of Oral Biomedicine Ministry of Education, Hubei Key Laboratory of Stomatology, School & Hospital of Stomatology, Wuhan University, Wuhan, China; 2 Department of Oral and Maxillofacial Surgery, School & Hospital of Stomatology, Wuhan University, Wuhan, China; 3 Department of Dental Implant Center, Beijing Stomatological Hospital, Capital Medical University, Beijing, China; 4 Laboratory for Clinical Medicine, Capital Medical University, Beijing, China; 5 Department of Oral and Maxillofacial Surgery, State Key Laboratory of Oral Diseases, National Center for Stomatology, National Clinical Research Center for Oral Diseases, West China Hospital of Stomatology, Sichuan University, Chengdu, China; 6 Department of Oral Surgery, College of Stomatology, National Clinical Research Center for Oral Diseases, Shanghai Key Laboratory of Stomatology, Shanghai Ninth People’s Hospital Affiliated to Shanghai Jiao Tong University School of Medicine, Shanghai, China; 7 Affiliated Stomatological Hospital of Nanchang University, Nanchang, China; 8 School of Stomatology, The Fourth Military Medical University, Xi’an, China; 9 Department of Oral and Maxillofacial Surgery, Peking University School and Hospital of Stomatology, Beijing, China; 10 National Center of Stomatology and National Clinical Research Center for Oral Diseases and National Engineering Research Center of Oral Biomaterials and Digital Medical Devices, Beijing, China; 11 Department of Stomatology, School of Stomatology, The Third Affiliated Hospital, Xi’an Medical University, Xi’an, China; 12 Department of Oral and Maxillofacial Surgery, School and Hospital of Stomatology, Jilin University, Changchun, China; 13 Jilin Provincial Key Laboratory of Tooth Development and Bone Remodeling, Jilin University, Changchun, China; 14 Affiliated Stomatological Hospital of China Medical University, Shenyang, China; 15 School of Stomatology, China Medical University, Shenyang, China; 16 The First Affiliated Hospital of Sun Yat-sen University, Guangzhou, China; 17 Hospital of Stomatology, Sun Yat-sen University, Guangzhou, China; 18 West China Hospital of Stomatology, Chengdu, China; 19 Shanxi Medical University School and Hospital of Stomatology, Taiyuan, China; 20 Shanghai Ninth People’s Hospital Affiliated to Shanghai Jiaotong University, School of Medicine, Shanghai, China; 21 School/Hospital of Stomatology Lanzhou University, Lanzhou, China; 22 School and Hospital of Stomatology, Fujian Medical University, Fuzhou, China; 23 Nanjing Stomatological Hospital, Nanjing, Jiangsu China; 24 Affiliated Hospital of Medical School, Nanjing University, Nanjing, China; 25 The Affiliated Hospital of Stomatology, School of Stomatology, Zhejiang University School of Medicine, Hangzhou, China; 26 Key Laboratory of Oral Biomedical Research of Zhejiang Province, Hangzhou, China; 27 Stomatological Hospital Affiliated Xiamen Medical College, Xiamen, China; 28 Department of International Medical Center, Beijing Stomatological Hospital, Capital Medical University, Beijing, China; 29 Beijing Laboratory of Oral Health, Capital Medical University, Beijing, China

**Keywords:** Third molar removal, Coronectomy

## Abstract

The third molar is the most developmentally delayed of the permanent teeth and has the highest incidence of pericoronitis and odontogenic space infections. Impacted third molars significantly increase the risk of periodontitis, dental caries, and external root resorption of adjacent second molars. Third molars are associated with complex surgical procedures, and treatment decisions and clinical management of third molars in this context remain controversial. This expert consensus was generated by oral surgeons and related specialists, who synthesized the current evidence-based literature and contemporary clinical practices. This consensus addresses critical considerations: the developmental trajectory and impaction characteristics of third molars, clinical and radiographic examinations of third molars, classification of impacted third molars, adverse effects of impacted third molars on oral health, indications for extraction, preoperative preparation for impacted third molar removal, anesthetic choices for impacted third molar surgery, recommended surgical protocol for impacted third molar removal, application of implant materials in alveolar sockets, management of common severe complications in impacted third molar extraction, and functional utilization of impacted third molars. Based on a comprehensive expert deliberation, this consensus provides evidence-based clinical guidance and standardized protocols for dental practitioners in the context of third molar management and therapeutic decision-making.

## Introduction

Third molars, commonly referred to as wisdom teeth, are the last teeth to develop and mature in humans. They exhibit the highest rate of impaction, cause the greatest damage to adjacent tissues, and present some of the most complex challenges in clinical management, often accompanied by considerable treatment controversies. Due to their late development and the limited space in the jaw, third molars are prone to abnormal eruption patterns, morphological variations, and pathologies such as dentigerous cysts.^1^ These irregularities frequently lead to complications, including impaction, pericoronitis,^2^ infection, delayed eruption, and disease progression,^3^ and increase the risk of periodontitis, dental caries, and external root resorption of adjacent second molars.

The developmental and eruptive patterns of third molars vary substantially among individuals. Patients’ perceptions and concerns regarding their third molars are influenced by factors such as age, geographic region, cultural practices, and customs. Similarly, clinicians’ management strategies depend on their educational background, specialty, and technical expertise.^4^ Such discrepancies may result in delayed or inappropriate interventions, leading to irreversible sequelae, unnecessary iatrogenic injury, or complications from overtreatment. This expert consensus on third molar health management aims to provide evidence-based recommendations to support standardized and well-informed clinical decision-making.

## Developmental trajectory and impaction characteristics of third molars

The development and formation of third molar tooth germs vary across populations and are influenced by factors such as sex, ethnicity, geographic region, and socioeconomic status. In Chinese individuals, mandibular third molar germs typically begin to form between the ages of 5–6 years and are initially identifiable on radiographs as shallow crypts located at the junction between the mandibular ramus and body.^5^ Calcification becomes evident around ages 7–9, indicating the initiation of crown development. During this stage, as the mandible grows rapidly, the tooth germ tends to migrate deeper into the mandibular body, with its long axis often tilting obliquely or even horizontally relative to adjacent teeth.

**Fig. 1:**
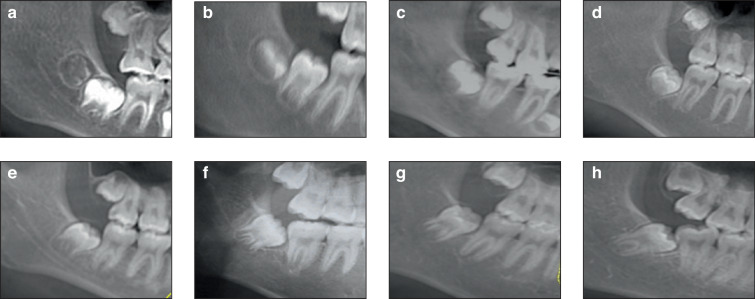
Radiological image of the anatomical development of the inferior third molar. The developmental stages include **a** the formation of the interdental sulcus; **b** development of the crown at the 1/3 stage; **c** development of the crown at the 2/3 stage; **d** completion of basic crown development; **e** early formation of the root; **f** development of the root at the 1/3 stage; **g** development of the root at the 2/3 stage; and **h** complete development of the root

By approximately 12 years of age, an individual’s crown formation is usually complete. Cervical formation occurs around ages 13–14, at which point the tooth germ remains beneath the occlusal plane, and the angulation between the germ and adjacent teeth begins to decrease. Simultaneously, root development commences extracapsularly. By ages 15–16, the roots reach approximately one-third of their final length, and the germ becomes radiographically visible distal to the roots of the second molars. Between the ages of 17–18, the root approaches two-thirds of its full length, the angulation continues to decline, and the crown begins to erupt into its final position. Complete root formation is typically achieved between ages 19 and 20. In healthy individuals, third molar development is delayed, with full maturation or eruption not occurring until approximately age 24.^6^ Maxillary third molars follow a broadly similar timeline, although females generally exhibit earlier development.^7^ Figure 1 presents a comprehensive anatomical illustration of the developmental trajectory of the mandibular third molar, emphasizing key milestones from the initiation of interdental sulcus formation to the completion of root development. Each stage provides insights into the morphological and biological processes underlying tooth maturation, which are crucial for understanding the functional and pathological characteristics of teeth in various clinical contexts.

Due to their late development and spatial limitations within the jaws, third molars are susceptible to anomalies in the eruption direction, crown morphology, size, and root number or length. These abnormalities frequently result in impaction and delayed maturation. The prevalence of impacted third molars varies significantly across studies and is influenced by the geographic region, ethnicity, and dietary habits. Impaction is more common among females, whose reported prevalence is 1.16–1.5 times higher than that among males.^6,8^ Patients typically present with symptoms of third molar impaction during early to mid-adulthood, particularly between the ages of 20–40 years.^9^

Adolescents exhibit a particularly high rate of mandibular third molar impaction, which varies from 69.1% to 95.2% of cases with mesioangular inclination.^10^ With aging, the proportion of median-position impactions increases, whereas the proportion of low-position impactions decreases, with vertical impactions being the most prevalent, accounting for up to 42%.^6,10^

A study of 13 168 individuals aged 17–22 years in central China reported a mandibular third molar impaction rate of 70.3%, with mesioangular impactions accounting for 58.5% of the cases. These findings emphasize the clinical importance of impaction, which may lead to complications such as pericoronitis, infection, and delayed eruption because of the limited space in the jaw and the late development of these teeth.

## Clinical and radiographic examinations of third molars

The evaluation of mature third molars involves both clinical and radiographic examinations, with consideration given to the patient’s local oral condition and overall systemic health status.

### Clinical examination

The key aspects of the clinical examination include the following:

Evaluation of facial symmetry and the presence of swelling, trismus, and regional lymphadenopathy.

Determination of third molar visibility, the extent of soft tissue coverage, and classification of the impaction type.

Assessment of the tooth structure for caries, fractures, or mobility and identification of percussion or periodontal tenderness.

Examination of adjacent teeth, including their alignment and occlusal relationship with the opposing dentition.

Gingival health is evaluated by examining the surrounding tissue of adjacent teeth for changes in color, texture, erythema, suppuration, fistulas, or ulcers.

### Radiographic examination

A radiographic evaluation plays a crucial role in determining the eruption status, impaction classification, anatomical relationships, and potential indications or contraindications for extraction. Common imaging modalities include periapical radiographs, panoramic radiographs, and cone-beam computed tomography (CBCT).

#### Periapical radiographs

Periapical radiographs can provide detailed information regarding the long-axis orientation and inclination of the third molar, periapical pathology, surrounding alveolar bone, and condition of adjacent teeth. They provide preliminary insights into the relationship between the mandibular third molar and the mandibular canal, as well as the proximity of the maxillary third molar to the maxillary sinus. This technique involves a low radiation dose and is relatively simple to perform. However, it may cause discomfort, particularly in posterior regions, and some patients—especially those with a strong gag reflex—may be unable to tolerate this examination. Furthermore, its limited field of view often fails to fully capture the relationship between third molars (particularly mandibular molars) and adjacent anatomical structures. As a two-dimensional imaging method, it presents overlapping structures, which can compromise spatial accuracy. The superimposition of structures in this two-dimensional imaging method may compromise the spatial interpretation. Thus, periapical radiography is most suitable for the preliminary assessment of partially erupted third molars under favorable periodontal conditions.

#### Panoramic radiographs

Panoramic imaging offers a broad overview of the dental arches and most maxillofacial structures, enabling an assessment of the eruption direction, depth, angulation, and anatomical relationships of third molars. It is well tolerated by most patients and clearly displays the mandibular canal trajectory, making it a standard imaging modality for evaluating third molars. However, similar to periapical radiographs, panoramic images are two-dimensional and cannot accurately depict three-dimensional spatial relationships. Additionally, image distortion and magnification errors (approximately 3%–10%) may compromise the evaluation of the root number, morphology, and proximity to critical structures such as the mandibular nerve canal. Therefore, although panoramic radiographs are highly useful for initial screening, supplementary imaging—particularly CBCT—is recommended in complex or high-risk cases.

#### Cone-beam computed tomography (CBCT)

CBCT provides high-resolution, three-dimensional visualization of third molars and their anatomical surroundings, thus facilitating precise assessments of their relationships with adjacent teeth, the mandibular canal, the maxillary sinus, and other key structures. It also facilitates the identification of pathology in adjacent tissues and a full evaluation of both jaws. CBCT is indispensable for advanced surgical planning, including digital guides, navigation systems, and robotic-assisted extractions. However, its use is associated with higher costs and greater radiation exposure than conventional radiography. CBCT is particularly valuable for evaluating third molars with suspected pathology or complex anatomical relationships, and it is essential in preoperative planning for high-risk extractions.

If a patient already presents with well-acquired spiral computed tomography (FBCT) images, an additional CBCT examination is generally not needed. The existing FBCT can provide sufficient anatomical detail for the assessment of impacted third molars, including their relationships with adjacent structures. Therefore, performing an additional CBCT would not offer a substantial diagnostic benefit and would expose the patient to unnecessary radiation. Consequently, the use of FBCT alone is considered adequate for preoperative planning in such cases.

## Classification related to impacted third molar extraction

Due to discrepancies between jaw development and eruption space, as well as their late developmental timing, most third molars become impacted, except for a minority that erupts normally. Classification methods include the Winter classification and Pell & Gregory classification, which are widely used clinically.

### Winter classification

Categorizes impactions by the axial position: vertical, mesioangular, horizontal, inverted (distoangular for upper molars), distoangular (a common terminology for lower molars), lingual/palatal, and buccal impaction. Mesioangular impaction is the most common clinical presentation. This classification guides the direction of surgical dislodgment and marginally aids in assessing the difficulty of the procedure.

### Pell & Gregory classification

Vertical categorization relative to the second molar occlusal plane/cementoenamel junction, level A impactions dominate clinically:

- Level A (shallow)—Third molar crown level with/above the second molar occlusal plane;
- Level B (moderate)—Below the second molar occlusal plane but above the second molar cervical line;
- Level C (deep)—Third molar positioned below the second molar cervical line.

Horizontally categorized by the ramus relationship, Class Ⅱ is the most prevalent class and facilitates the evaluation of surgical difficulty and planning:

- Class Ⅰ: Adequate space between the third molar and anterior ramus border;
- Class Ⅱ: The third molar is occupying part of the ramp space;
- Class Ⅲ: The third molar is entirely within the mandibular ramus.

Both systems have limitations in terms of their ability to fully describe impaction status and extraction resistance. Therefore, they are often combined in clinical practice.^11,12^

### Pell & Gregory classification modified by Rui Sun and Ji-hong Zhao

Category C includes all mandibular third molars below the cervical line of the second molar, which encompasses a wide range and does not accurately reflect the impaction depth or surgical difficulty. Teeth with a linear distance from the second molar exceeding the tooth length or located below its apical one-third are further classified as Category D teeth to improve precision. This subclassification more accurately represents the impaction depth and better predicts the complexity of extraction.

### Archer classification

Based on the relative depth of the maxillary impacted third molar within the bone, four categories have been proposed:

Level A—The lowest point of the crown of the maxillary impacted third molar is at the same level as the occlusal plane of the adjacent second molar.

Level B—The lowest point of the crown is located between the occlusal plane and the cervical line of the second molar.

Level C—The lowest point of the crown lies between the cervical line and the apical third of the second molar.

Level D—The lowest point of the crown is positioned at or above the apical third of the second molar.

**Fig. 2:**
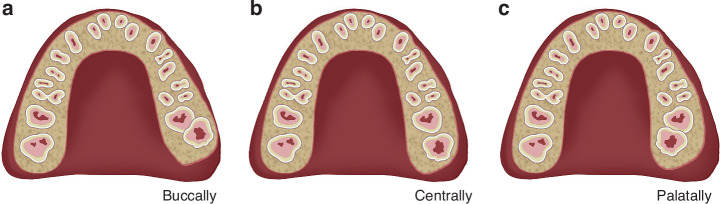
Buccal‒palatal crown positions between the maxillary third molar and second molar. The measurement starts from the bulbosity of the crown of the adjacent second molar because it affects the difficulty and accessibility of using an elevator and rotary handpiece in the buccal or palatal direction. Tooth #28 is shown as an example: **a** partial buccal position, **b** middle/central position, and **c** partial palatal position

While the classification of maxillary impacted third molars is often adapted from the Pell and Gregory system used for mandibular third molars, it does not include the traditional Class Ⅰ to Ⅲ categories. In an attempt to address this limitation, Chinese scholars have proposed a classification scheme based on the position of the buccopalatal crown of the maxillary third molar relative to the adjacent second molar, designating the tooth as being buccally impacted, centrally impacted, or palatally impacted. This refined system provides greater clinical relevance, as it can guide surgical planning; in particular, centrally or palatally impacted cases may benefit from a palatal approach, thereby facilitating extraction and reducing the risk of traumatic injury to the labial mucosa^13^ (Fig. 2).

## Adverse effects of impacted third molars on oral health

Third molars, owing to their physiological characteristics, such as an insufficient eruption space, abnormal eruption direction, and delayed eruption time, are more prone to impaction than other permanent teeth; thus, these teeth are more likely to cause complications affecting themselves and adjacent tissues. Common clinical complications are listed below.

### Pericoronitis

Pericoronitis is most common in the mandibular bone around third molars (82.41% incidence^1^), typically manifests with local symptoms such as swelling, pain, and limited mouth opening, and in severe cases, it may be accompanied by systemic manifestations. M3Ms with impaction level A were more likely to be accompanied by pericoronitis than those with impaction level B, whereas M3Ms with impaction class Ⅱ had a greater risk of pericoronitis than those with impaction class Ⅰ.^14^ A soft tissue inflammatory response surrounding partially erupted or impacted third molars most commonly involves mandibular third molars (reported incidence: 82.41%)^15^. Level A impactions are associated with a greater incidence of pericoronitis than level B impactions, and class Ⅱ impactions present a greater risk than class Ⅰ impactions.

### Infection of the maxillofacial space

Impacted third molars are responsible for 50%–70% of fascial space infections. Pericoronal infections may spread via potential pathways to the submandibular, parapharyngeal, and pterygomandibular spaces. Untreated cases may progress to multiple space infections requiring urgent treatment because of the risks of Ludwig’s angina, intracranial infections, or septicemia.^15^

### Osteomyelitis

Osteomyelitis occurs in approximately 6% of cases involving chronic pericoronitis because of the deep spread of infection through the alveolar bone^16^.

### Adjacent tooth/crevicular effects

The anatomical position of third molars creates plaque-retentive niches, turning their periodontal tissues into reservoirs of pathogenic microorganisms, which can compromise the periodontal health of adjacent second molars.^17^ Persistent food impaction between the third and second molars further exacerbates periodontal destruction.^16^ Consequently, second molars adjacent to third molars exhibit a significantly higher prevalence of periodontitis than those without third molars.^18^ Moreover, third molars may contribute to distal interproximal caries of second molars, with approximately 19.5% of patients with impacted third molars presenting distal caries in adjacent second molars.^17^ Mesioangular or horizontal impactions can additionally exert sustained pressure on the roots of second molars, leading to external root resorption.^17^

### Odontogenic cysts and tumors

Impacted third molars are a significant potential cause of jaw cysts and tumors, with approximately 5% of patients with impacted third molars developing associated jaw cysts or tumors.^19^ These pathologies account for significant mandibular morbidity.^20^

### Temporomandibular disorders (TMDs)

The impaction and abnormal eruption of mandibular third molars may contribute to the onset or aggravation of temporomandibular disorders (TMDs) through multiple mechanisms. Abnormal eruption patterns can cause condylar displacement and impair the function of pterygoid muscles, leading to joint dysfunction. In adults, impacted third molars may exert pressure on adjacent teeth, alter the curve of Spee, and produce occlusal interference that predisposes patients to TMD. Similarly, ectopic eruption may induce premature occlusal contact and occlusal trauma, further exacerbating joint symptoms. Periodontal pain associated with impacted third molars often leads to unilateral mastication, resulting in masticatory muscle overload and functional imbalance. Moreover, acute or recurrent pericoronitis can induce trismus, which increases mechanical loading of the temporomandibular joint and aggravates existing TMD manifestations. Clinical and imaging studies have confirmed that pericoronitis-related inflammation and pain may radiate to the TMJ region, often manifesting as restricted mouth opening, ear pain, or masticatory muscle spasm, and may even be misdiagnosed as primary TMD without a careful differential diagnosis^21,22^

## Indications for extraction

Treatment decisions balance tooth viability with risks and benefits after a comprehensive assessment. All the extraction indicators should favor clinical outcomes.

### Definite pathologic indicators

- Third molars involving recurrent pericoronitis/space infections;
- Carious teeth or those causing adjacent caries;
- Third molars causing occlusal interference (overeruption)/chronic food impaction;
- Periodontal damage to second molars;
- Odontogenic cyst/tumor association;
- Moderate/severe adjacent root resorption;

### Therapeutic extraction needs

- Orthodontic demands (space needs, molar uprighting, vertical adjustment);
- Third molars are located on the planned orthognathic osteotomy lines;
- TMD management protocols;
- Pretreatment clearance prior to radiotherapy/chemotherapy/immunotherapy;
- Preemptive removal before pregnancy due to an increased risk of pericoronitis with deep periodontal involvement.

### Prophylactic removal of symptomatic but potentially risky third molars^23^

- Partial eruption of medially or horizontally impacted third molars;
- Soft tissue-impacted third molars that have not yet erupted;
- Complete bony-impacted third molars if the distal alveolar bone of adjacent teeth is deficient up to the crown of the impacted third molar or if the adjacent tooth roots show mild external resorption.

Notably, these potentially risky third molars do not inevitably lead to adjacent tooth decay, loosening, loss, or other complications. Therefore, the decision to remove them should account for the patient’s preferences, the clinician’s expertise, and potential surgical complications. For patients who are reluctant to agree to removal (e.g., over 35 years of age with complete bony impaction), observation may be advised if the degree of surgical risk is high.

## Preoperative preparation for impacted third molar removal

Owing to the complexity and risks of third molar extraction, specialized preparation is required beyond standard tooth removal protocols:

- Imaging, including mandatory preoperative X-rays and CBCT, if necessary.

- Laboratory tests include blood analysis (including coagulation) and additional tests (glucose, ECG, and liver/kidney function) as needed.

- An assessment of the patient’s condition should ensure good health and avoid surgery during menstruation or pregnancy (specifically in the first/last trimesters). For the management of third molars associated with anticoagulant oral therapy, care is required for patients with a complicated medical history, and a comprehensive multidisciplinary evaluation is needed.^24^
- Premedication consists of administering prophylactic antibiotics 30–60 min prior to surgery, along with NSAIDs, to provide preemptive analgesia and manage postoperative pain.^25,26^

Sterile supplies, including surgical instruments, drapes, and dressings, should be prepared.

In terms of instruments, micropower systems are suggested. At present, reusable pneumatic handpieces are commonly used in clinical practice in China. The use of piezosurgery or a handpiece with saline irrigation is recommended for bone removal and tooth sectioning to prevent infection. Saline or sterile water should be available for irrigation and cooling.

- Regarding tooth socket filling materials, in most cases of extraction, bone graft substitutes or socket hemostatic agents are not necessary. Bone graft substitutes and socket hemostatic agents should be prepared as needed.

## Anesthetic choices for impacted third molar surgery

Local anesthesia is routinely employed for impacted third molar extraction. The most commonly used agents in China include lidocaine, articaine,^27^ and mepivacaine.^28^

For maxillary impacted third molars, posterior superior alveolar nerve block and greater palatine nerve block are frequently applied. Because the surrounding bone is relatively less dense, with thinner cortical plates and an abundant vascular supply of soft and hard tissues, local infiltration anesthesia with articaine is often preferred.^29^ This approach not only provides satisfactory anesthetic effects but also effectively reduces intraoperative bleeding. Importantly, even for deeply impacted maxillary third molars, local infiltration with articaine can achieve effective anesthesia. At such depth, the diffusion pattern of the anesthetic essentially mimics the effect of block anesthesia, thereby ensuring sufficient analgesia.

For mandibular impacted third molars, the surrounding bone is denser, with thicker cortical plates and the presence of internal and external oblique ridges. Therefore, inferior alveolar, buccal, and lingual nerve blocks are the first-line anesthetic techniques. Alternatively, multiple-site infiltration with a sufficient volume of articaine can also achieve satisfactory anesthesia.^30^

With respect to anxiety management, nitrous oxide inhalation or painless injection devices may be used to reduce patient discomfort.^31^ In patients with severe dental anxiety, high surgical difficulty, or simultaneous extraction of multiple impacted teeth, general anesthesia may be considered.^32^

## Recommended surgical protocol for impacted third molar removal

### General principles

- Resistance analysis: Perform presurgery planning via clinical and radiographic evaluations (tooth orientation, root morphology, and bone resistance).
- Safety: Minimize damage to adjacent tissues, nerves (inferior alveolar, lingual), and structures (maxillary sinus).
- Minimally invasive: Prioritize tooth splitting over excessive bone removal; preserve alveolar crest integrity.

- Sterility: Asepsis is a fundamental principle of surgical procedures. Owing to the unique environment of the oral cavity, absolute asepsis is challenging to achieve during surgery.
  Therefore, maintaining asepsis during surgery is crucial. In accordance with the “Standard for Infection Management in Dental Outpatient Clinics” (WS/T842-2024) issued by the National Health Commission, the water used for cooling and irrigation during oral surgery must be sterile. For impacted third molars that require incisions, flap elevation, and bone removal, a piezosurgery unit and handpiece should be used with sterile saline, sterile water, or distilled water for irrigation and cooling to reduce the risk of postoperative surgical wound infection.^26,33^ Since the air and water pathways of high-speed dental handpieces cannot be fully sterilized, strict infection control measures should be implemented during and after their use to prevent postoperative infection.

### Surgical techniques for the extraction of impacted third molars

(1) Indications: These techniques are appropriate for most third molar extraction procedures.

(2) Incisions: Common incisions include the following:

- Retromolar incision (for Pell & Gregory level A)
- Retromolar incision extending to the buccal gingival sulcus of first/second molars (level B)
- Retromolar incision with mesial–vertical release (levels C and D). Palatal/lingual incisions may be added for correspondingly positioned impactions.

(3) Mucoperiosteal Flap Elevation: This technique predominantly involves buccal flap elevation with 2–3 mm extension beyond the intended bone removal area. The lingual tissues should be preserved to avoid injuring the lingual or palatine nerves and vasculature.

(4) Window osteotomy: For osseously impacted third molars, proper window osteotomy should be performed to expose the impacted tooth and eliminate bone resistance. The bone removal range should adequately expose the crown of impacted third molars, preferably distal to the second molar. For deeper (level C or D) impacted teeth lingual/buccal to the second molar, at least 5 mm of alveolar crest bone height should be maintained on the facial and lingual aspects of the second molar to preserve periodontal health. For Pell & Gregory class Ⅲ horizontally impacted third molars largely embedded in the mandibular ramus, significant bone removal is needed to eliminate resistance.

(5) Tooth Sectioning: Sectioning effectively resolves resistance, reduces trauma, and minimizes complications. Multiple techniques exist, such as mesiodistal root separation, coronal division, cervical crown‒root sectioning, tri-segment splitting, or T-shaped sectioning. Operators should flexibly apply these methods based on the impaction patterns, resistance locations, and dislocation paths, in which context, minimal bone removal and minimal complications should be prioritized. Multiple sectioning methods or repeated divisions may be combined. In maxillary third molar extraction with limited visual/operating space, sectioning is less frequent, although single-section division is sufficient when necessary.

(6) Tooth Extraction/Displacement: After resistance is reduced through osteotomy and sectioning, appropriate minimally invasive elevators or forceps should be selected to sequentially retrieve the crown and roots. Continued retention indicates residual resistance, necessitating the reassessment of retention points and an additional reduction in resistance before extraction.

(7) Socket Management: After extraction, inflammatory granulation tissues/residual follicles should be curetted from the socket. Residual tooth fragments/bone debris should be removed, followed by irrigation with saline or sterile water, the achievement of hemostasis, and the optional application of bone substitutes or CGF.

(8) Wound Closure: Wound closure involves thorough suturing for flap wounds; in particular, the distal cervical region of the second molars should be sealed to prevent food/saliva infiltration, which affects healing. Loose suturing should be used for sockets with questionable hemostasis to facilitate drainage. Polytetrafluoroethylene polymer sutures are superior to silk sutures for reducing bacterial biofilms in both aerobic and anaerobic evaluations after tooth surgery.^34^

### Characteristics of third molar germ extraction

Based on the developmental and maturational characteristics of third molars in the Chinese population, third molars in individuals younger than 18 years, with incomplete root development and unerupted crowns, are generally defined as being in the “germ stage”.^35^ Due to the considerable individual variation during this stage and the fact that the germ stage may last 13–15 years, unexpected changes^35–39^ can occur in the germ and surrounding tissues. Therefore, appropriate health management must be implemented during this period. For third molar germs that have been identified as potentially harmful, regular monitoring and assessments should be conducted,^40^ with timely intervention when problems arise.^41^

The procedure of third molar germ extraction is similar to the basic surgical technique described above, with the following distinctive features:

(1) Patients are generally younger, exhibit poorer compliance, and experience greater preoperative anxiety. Therefore, surgeons should establish effective communication to gain the patient’s trust prior to surgery.

(2) Appropriate sedation should be administered preoperatively and intraoperatively to ensure adequate local anesthesia. The operator must be technically proficient and perform maneuvers gently.

(3) Younger patients tend to have third molar germs positioned more buccally, with relatively thicker lingual/palatal bone walls.^42^ The presence of interposed bone between the germ and anatomical structures (mandibular canal/maxillary sinus) typically reduces the intraoperative risk of damage to the inferior alveolar nerve, lingual nerve, or maxillary sinus.^43^

(4) Root development of the third molars is incomplete at this stage, resulting in less resistance during extraction. While the lower mineralization level of the tooth germ facilitates faster sectioning, the mobility of the dental germ within the follicle complicates the sectioning process.

(5) Third molar germ extraction causes minimal trauma, results in milder postoperative complications, and enables faster tissue healing.^44^

### “Root Removal First” strategy for impacted third molar extraction^45^

**Fig. 3:**
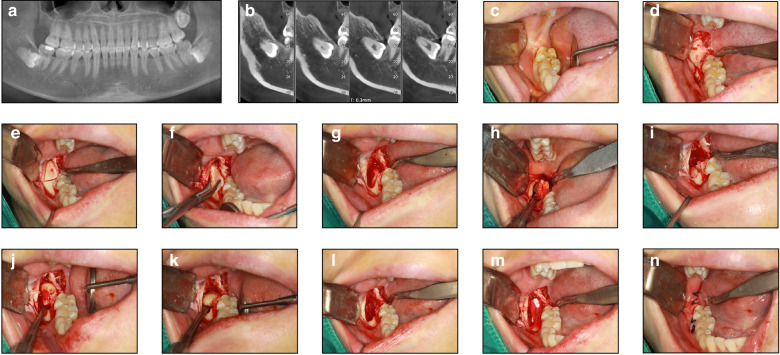
The extraction of tooth #48 via the “root removal first” strategy. **a**, **b** Preoperative CBCT image. Tooth #48 is in a horizontal position, and the crowns are located below the cervical line of tooth #47. **c** Tooth #48 is invisible in the oral cavity. **d** Exposed bone surface of the IMTM. **e**, **f** Bone window creation. **g** Revealed root of tooth #48. **h** Impacted tooth separation and root removal. **i**–**k** Crown removal. **l** Irrigation of the alveolar socket. **m** Placement of a collagen plug sponge into the tooth socket. **n** Sutured wound

This strategy is indicated for deeply embedded (level C or D), mesioangular or horizontal impacted mandibular third molars, where the crown closely abuts the second molar root or the roots lie near the inferior alveolar nerve. The distal bone to the second molar is removed to expose the third molar neck and part of the roots. The tooth is sectioned along the long axis at a 60° angle from anterosuperior to posteroinferior, and the root is elevated first. The crown is then pushed apically and removed, preserving the periodontal health of the second molar (Fig. 3).

### Submaxillary sinus membrane space approach for the extraction of maxillary impacted third molars^46^

**Fig. 4:**
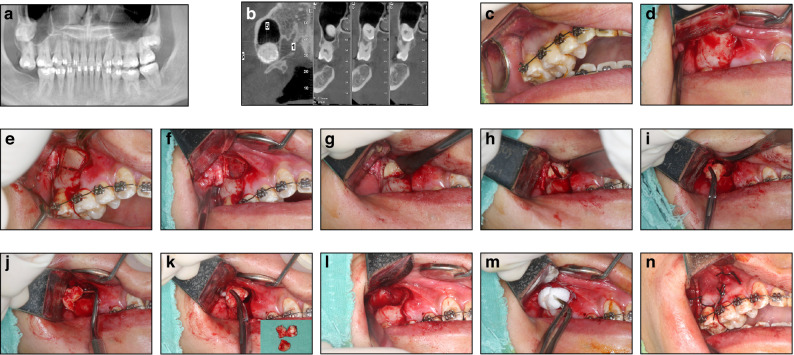
Extraction of an impacted maxillary third molar adjacent to the maxillary sinus via the submaxillary sinus membrane space approach. **a**, **b** CBCT images indicating the position of the impacted third molar. **c** The molar is invisible in the oral cavity. **d** Design of the incision and elevation of the mucoperiosteal flap. **e** The bony window created via piezosurgery. **f** Elevation and reflection of the buccal bone flap to reveal the underlying maxillary sinus membrane. **g** Elevation of the maxillary sinus membrane resulting in tooth exposure. **h**–**k** Removal of the impacted tooth in pieces following tooth sectioning through the bony window. **l** Intact maxillary sinus membrane. **m** Copious irrigation and placement of a collagen plug sponge into the sub-MSM space for hemostasis with the aim of preventing oroantral communication. **n** Primary suturing of the wound

This approach is indicated for level C or D maxillary impacted third molars with significant sinus protrusion. A 10×10 mm^2^ trapezoidal bone window is created above the apex of the second molar using ultrasonic osteotomy, and the sinus mucosa is gently elevated to form a submucosal space. The impacted tooth is exposed and removed through this space, minimizing the risk of sinus membrane injury, tooth displacement into the sinus, or damage to adjacent roots (Fig. 4).

### Orthodontic traction followed by the extraction of impacted mandibular third molars^47^

**Fig. 5:**
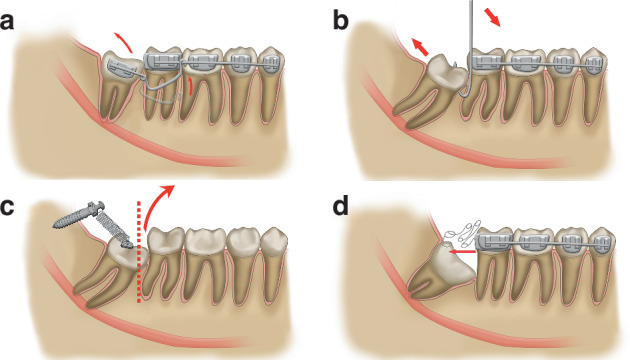
Orthodontic traction implementation. **a** One-step approach for vertical impaction. **b** An orthodontic stainless steel rectangular wire with a complete unequal-arm U-loop. **c** Placement of an orthodontic mini-implant in the ramus region, followed by the connection of a tension spring between the mini-implant and the impacted tooth. **d** Step 1 of the two-step procedure: bonding of a band to the adjacent tooth, followed by welding of a push spring fabricated from an orthodontic stainless steel round wire to the distal side of the band. Bonding of a stop loop with a mesial opening to the occlusal–distal surface of the impacted molar

This approach is applied to vertical, mesioangular, horizontal (axial angle <30°), or distoangular (axial angle <110°) mandibular third molars in close contact with the mandibular canal. Orthodontic forces gradually displace the tooth to separate the roots from the canal before extraction, reducing the risk of inferior alveolar nerve injury and promoting distal bone formation near adjacent teeth. This procedure requires the participation of an orthodontist or a general dentist with specialized orthodontic training (Fig. 5).

### Coronectomy of the impacted mandibular third molar^48–50^

**Fig. 6:**
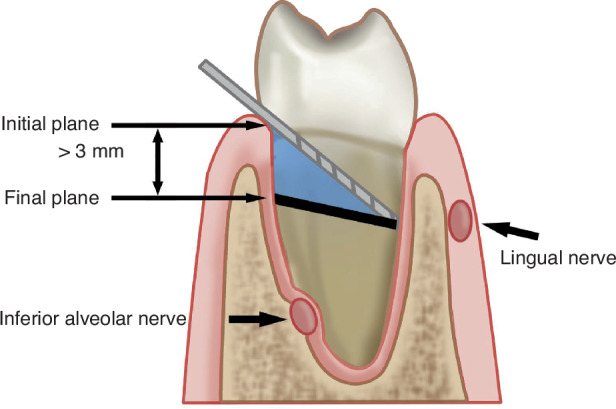
Coronectomy of the impacted mandibular third molar. The distance between the alveolar crest and the final plane should be greater than 3 mm

This approach is indicated when mandibular third molar roots are closely associated with the mandibular canal or when roots are bulbous or curved, posing a high risk of nerve injury.^51^ Only the crown is removed, retaining part of the roots within the jaw. The crown section is located at or below the pulp chamber floor and 3–5 mm below the crestal bone margin.^52^ Postoperative monitoring includes a CBCT evaluation to assess the root position, bone coverage, and potential for secondary root removal, if necessary.^53–55^ Coronectomy reduces the risk of inferior alveolar nerve injury and postoperative pain while maintaining the periodontal health of the second molar (Fig. 6).

### Application of digital technology in impacted third molar extraction^56–61^

Digital technologies have become valuable tools for complex or high-risk impacted third molar extractions, enhancing surgical precision, reducing the surgical risk, and minimizing complications. Digital guide-assisted bone window preparation ensures accurate localization and bone removal, protecting critical structures such as the inferior alveolar nerve, maxillary sinus, and adjacent roots.^57^ Digital navigation systems^49^ and surgical robots further allow precise control of the tooth sectioning direction and depth, improving procedural safety. Although these technologies currently require additional preparation time and cost, they are particularly beneficial for high-risk patients and less experienced surgeons, with ongoing advancements expected to streamline their clinical application. Additionally, endoscope-assisted extraction improves the visualization of deep surgical sites, facilitating refined manipulation and safer removal of deeply impacted third molars where conventional direct vision and intraoperative imaging are limited. Collectively, these digital and endoscopic approaches contribute to improved surgical outcomes, reduced intraoperative trauma, and a lower incidence of postoperative complications.

### Clinical decision trees

**Fig. 7:**
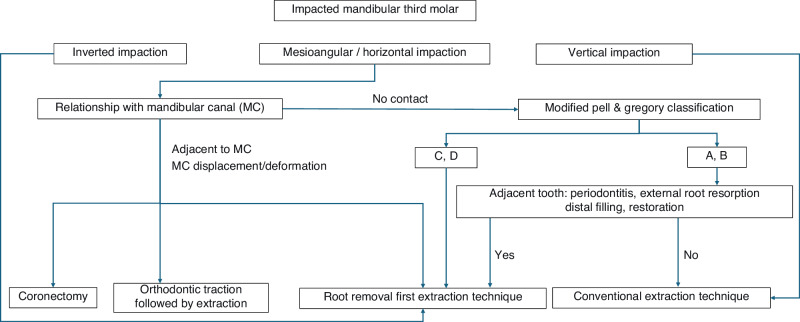
Selection of the surgical approach for mandibular third molars

**Fig. 8:**
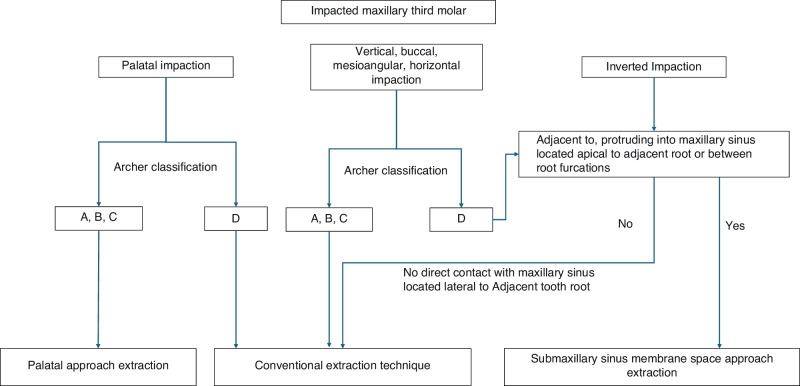
Selection of the surgical approach for maxillary third molars

The selection of the surgical approach for the mandibular (Fig. 7) and maxillary third molars (Fig. 8) is shown below. However, a consensus has not been reached on the extraction timing and use of digital technology. Managing special populations, including pregnant women, elderly patients, and individuals with systemic diseases, is challenging because of the complexity of their cases. Each requires a tailored treatment plan, as reliance on simplistic decision-tree models is insufficient.

## Application of implant materials in alveolar sockets

The extraction of impacted third molars involves flap elevation and bone removal, resulting in relatively greater surgical trauma, a prolonged operative time, and possible distal alveolar bone resorption adjacent to the second molar.^62^ Therefore, appropriate postextraction socket management is critical for promoting healing and preventing complications such as bleeding, infection, and periodontal breakdown of adjacent teeth.

### Placement of hemostatic materials

After the extraction of impacted third molars (particularly impacted mandibular third molars), varying degrees of bleeding may occur within the alveolar socket. Significant bleeding must be controlled. For mild bleeding (20–30 s without blood completely filling the socket), hemostatic materials are generally unnecessary. For moderate bleeding (socket filled with blood within 10–15 s), materials such as collagen sponges or absorbable hemostatic gauze can be placed in the socket to achieve effective hemostasis.^63,64^ For arterial bleeding, bone wax or iodoform gauze should be used for compression hemostasis. For special cases, a coagulation screening test is necessary.^65^ Cyanoacrylate tissue adhesive could replace silk sutures for the closure of surgical wounds due to its beneficial hemostatic effects on postoperative bleeding.^66^ The use of this adhesive may reduce postoperative oral disability, facial swelling, and erythema after tooth extraction, with increased average and medium pain when cyanoacrylate gel is applied to the blood clot and sutured to promote recovery for secondary closure.^67^

### Implantation of materials that promote alveolar bone healing

After a routine impacted third molar extraction, the alveolar socket usually achieves satisfactory healing without the use of bone-promoting materials. However, if a preoperative reduction in buccolingual and distal alveolar bone height around the second molar is present or if buccolingual crestal bone removal occurs during surgery, postoperative root exposure or deep periodontal pockets at the second molar may develop.^68^ The following bone-healing materials may be implanted in the socket to preserve the periodontal health of the second molar:

(1) Concentrated Growth Factor (CGF): As a third-generation platelet concentrate, CGF comprises fibrin gel embedded with growth factors that promote microvascular regeneration.^69–71^ Its 3D fibrous network structure supports cell adhesion, proliferation, tissue contour restoration, and regeneration.^72–74^ CGF can be implanted alone into the third molar socket. If buccolingual crestal bone loss at the second molar is significant, CGF should be mixed with bone substitute materials to restore the height of the alveolar crest.

(2) Bone Substitute Implantation: Implanting bone substitutes into the third molar socket after extraction effectively restores the buccolingual and distal alveolar bone height at the second molar, maintains the osteogenic space, guides bone regeneration, and reduces postoperative periodontal complications.^75,76^ Common substitutes include autologous bone, allografts, xenografts, and synthetic materials, which are ideally absorbable within 3–6 months.^77,78^ The forcing of substitutes into the mandibular canal should be avoided, tension-free soft tissue closure should be ensured, and membranes should be used if primary closure is challenging.

(3) Bone Lid Technique: For deeply impacted mandibular third molars requiring extensive buccal/distal bone removal near the second molar, a predesigned bone window (size/shape) is cut with the assistance of piezoelectric tools through cortical and partial cancellous bone. The harvested bone block, preserved in saline ( < 30 min), is reimplanted after extraction to restore the height of the alveolar crest and prevent periodontal complications. Stabilizing the reimplanted block by underlying tissue/material support is critical for success.^79,80^

## Clinical guidelines for the postoperative management of the extraction site of impacted third molars

### Postoperative follow-up and review

- Postoperative follow-up: Patients should be contacted via telephone or online communication on the second day after surgery to assess their general condition and the surgical site and to provide necessary health instructions.
- Complication management: Patients who experience active bleeding or other postoperative complications should be advised to return to the hospital promptly for a clinical evaluation.

Suture Removal and Early Healing Assessment: Soft tissue wounds generally heal within 7–10 days. A follow-up appointment should be scheduled during this period to evaluate wound healing, remove sutures, and examine adjacent teeth. Any abnormalities should be addressed in a timely manner.

- Bone-healing assessment: For patients with significant preoperative bone defects on the distal aspect of the adjacent tooth (second molar), a follow-up visit at approximately three months after the operation is recommended to assess alveolar bone healing through a clinical or radiographic examination. If healing is unsatisfactory, appropriate measures should be taken, or the patient should be referred to a periodontal specialist.

### Principles of antibiotic use

- Indications: Prophylactic antibiotics are generally not required for the uncomplicated extraction of impacted maxillary third molars in patients in good overall health.
- After complex extraction: If the procedure is complex, involves a prolonged operation time, or causes significant tissue trauma, the risk of postoperative infection increases, and prophylactic antibiotics are recommended.
- Regimen selection: A combination of β-lactam antibiotics and nitroimidazole agents may be used for prophylaxis. The route of administration (oral or intravenous) should be determined based on the patient’s specific condition, typically for a duration of 3–5 days.

## Management of common severe complications of impacted third molar extraction

Due to factors such as the position of impacted third molars, the systemic or local tissue health of patients, surgical instruments, or the operator’s clinical skills, severe complications may arise.^81^ Once serious complications occur, corresponding treatment should be provided promptly if conditions permit. If managing complications is difficult,^82^ timely referrals should be arranged while ensuring patient safety.

### Management of nerve injury

Impacted third molar extraction may lead to inferior alveolar nerve injury, lingual nerve injury, or, occasionally, greater palatine nerve injury. Most nerve injuries involve friction, contusion/laceration, or crush damage, with rare cases of complete transection. Patients with inferior alveolar or lingual nerve injuries typically experience significant numbness and require active treatment, whereas greater palatine nerve injuries often cause minimal discomfort.^83,84^

For contused or crushed nerves, the following steps should be considered:

- Administer glucocorticoids or sodium aescinate intravenously to reduce edema (1-week course).^85^
- Prescribe vitamin B₁ + vitamin B₁₂ or mecobalamin tablets to promote nerve recovery (3–6 months).
- Recommend low-level laser therapy.^86^
- Nerve anastomosis should be performed promptly for transected nerves.

### Management of root displacement into the mandibular canal^60^

Root fractures during the extraction of impacted mandibular third molars may occur because of the root curvature, proximity to the mandibular canal, or enlarged apices. During the process of fragment retrieval, careless maneuvers may push root fragments into the mandibular canal.

- For fragments <3 mm in length with a penetration of <1/3 of the canal diameter, where no neurological deficits are observed, close observation is necessary if retrieval is challenging.
- For fragments >3 mm in length, those penetrating ≥1/2 of the canal diameter, or those with neurological deficits, prompt removal is required.

Key principles for surgery include:

- Preoperative CBCT imaging for precise localization.
- Maintenance of clear visualization or the use of an endoscope during surgery. Piezoelectric ultrasonic devices should be used to create space around the fragment, followed by minimal-force extraction with microforceps or hemostats. Levering or rocking motions should be avoided to prevent secondary nerve injury or deeper fragment displacement.

Intraoral endoscopy (rigid or flexible) enhances visualization and minimizes trauma during fragment retrieval^60,61^:

- Rigid endoscopes offer high resolution and compatibility with ultrasonic instruments.
- Flexible endoscopes offer adjustable angles for improved exploration.

### Management of root displacement into the lingual space^87^

The lingual bone plate of impacted mandibular third molars is usually thin, and partial lingual bone plate loss may occur in some roots. During the extraction of impacted mandibular third molars, roots or even entire teeth may displace into the lingual soft tissue space. Teeth or roots entering soft tissue spaces should be retrieved whenever possible.

During mandibular third molar extraction, if a tooth or root “disappears” from the socket, CBCT should be performed to determine its position. If the entire tooth has shifted into the lingual space, it can often be palpated through the lingual mucosa. In an attempt to push the tooth upward, partial removal of the lingual bone plate may be necessary to extrude the tooth. If this attempt is unsuccessful or for displaced roots, a triangular incision can be made distal to the mesiolingual aspect of the second molar to reflect the lingual mucoperiosteal flap. Direct visualization allows the retrieval of the displaced tooth/root from the soft tissue space corresponding to the lingual bone plate defect using vascular forceps. Intraoral endoscopy increases safety and efficiency. The lingual nerve should be protected, and hemostasis should be ensured.

For overlooked teeth/roots in soft tissue spaces, the following steps are important:

- Strict follow-up should be provided with periodic CBCT.
- Immediate intervention is needed if displacement or inflammatory changes occur.

### Management of tooth displacement into the maxillary sinus^88^

The impacted maxillary third molars are often adjacent to the maxillary sinus, which increases the degree of displacement risk during extraction. Immediate retrieval is strongly advised.

Procedure:

- Confirm displacement via CBCT when a tooth/root “disappears” intraoperatively.
- Extend the buccal incision and flap reflection to expose the anterolateral sinus wall.
- Create an ~0.8 × 0.8 cm^2^ window in the anterolateral wall for suction-assisted retrieval.
- Irrigate and perform thorough aspiration of sinus fluids, particularly for patients with a history of sinusitis.
- Reposition and suture the mucoperiosteal flap over the window.
- Place the collagen sponge/CGF in the socket and achieve tight closure.

Retained sinus items require the following:

- Regular CBCT monitoring.
- Prompt management of sinusitis complications.

### Management of alveolar bone vascular hemorrhage

Uncontrolled bleeding from small vascular branches in the impacted third molar alveolus requires immediate attention.

For bleeding during extraction, the following steps are needed:

- Apply bone wax/temperature-sensitive gel to achieve hemostasis.
- Clear excess materials postextraction.
- Consider aborting the procedure if the hemorrhage recurs.

For postextraction alveolar bleeding, use bone wax or iodized gauze packing.

### Management of dry socket

Dry socket (alveolar osteitis) is a relatively common postoperative complication following third molar extraction, particularly following the removal of mandibular third molars. Pathologically, it results from exposure of the alveolar bone walls, leading to an infection of the socket. The hallmark clinical symptom is severe, persistent pain. Therefore, the core principles of managing dry socket are infection control and pain relief.

Initial clinical management involves the debridement of necrotic tissue and food debris from the socket, followed by irrigation with sterile saline and thorough suctioning. An iodoform gauze strip or minocycline hydrochloride can subsequently be placed in the socket and replaced every 3–5 days.^89^ When fresh granulation tissue or bleeding is observed within the socket, CGF can be applied to reduce the frequency of dressing changes and shorten the overall treatment course.^90^ During this period, adjunctive use of photobiomodulation therapy (PBMT) can effectively promote socket healing.^91^ For patients experiencing severe pain, appropriate analgesics should be administered.

## Functional utilization of impacted third molars

Impacted third molars or those without opposing teeth should be preserved as much as possible if they do not cause clinical symptoms. When adjacent molars or premolars require extraction, functional recovery can be achieved through autotransplantation or orthodontic movement of the third molar.

### Functional utilization of third molar autotransplantation^92^

Autogenous tooth transplantation involves transferring a tooth from one position to another within the same individual.^93–96^ Third molars are common donor teeth that are typically transplanted to replace missing first or second molars and, occasionally, to premolar regions.

A preoperative assessment (clinical examination and CBCT imaging) must evaluate transplant feasibility based on the following parameters:

- Root development that is ≥3/4 complete;
- A healthy tooth structure that can be extracted intact with minimal trauma;
- Adequate recipient alveolar bone volume;
- Compatibility between the donor tooth and the recipient site.

A digitized 3D donor tooth model and surgical guide should be created preoperatively. The recipient socket is prepared using the donor model after the target tooth is extracted. The third molar is minimally extracted to preserve periodontal ligament integrity and avoid root fracture and is then immediately transplanted to the recipient site (extracorporeal time ≤15 min) and stabilized. Root canal therapy may be performed 2–4 weeks after surgery.

The criteria for successfully autotransplanting third molars are listed below.^96,97^

Twelve-month post-operative verification of the following parameters is needed:

- Normal physiological mobility;
- A normal percussion tone;
- Absence of periodontal pockets/inflammation;
- Functional occlusion.

Radiographic confirmation of the following parameters is needed:

- Normal periodontal ligament space;
- No progressive internal/external root resorption;
- Hard lamina at the alveolar margin.

Current success rates in China have reached ~95%, demonstrating the effective functional utilization of impacted/non-functional third molars.^98–105^

### Functional utilization of orthodontic third molar mesialization^106–108^

Orthodontic movement preserves the functional potential of impacted/non-functional third molars. When neighboring first/second molars require extraction or lack a long-term prognosis, the selective extraction of compromised teeth allows third molar mesialization for functional substitution.

For malocclusion cases requiring premolar extraction to gain orthodontic space, the following point should be considered:

Adjusted therapy prioritizes the extraction of severely damaged first/second molars (with favorable third molars), thereby preserving intact premolars.

For non-orthodontic patients who require first/second molar extraction, the extraction of compromised molars with third molar mesialization preserves healthy natural dentition.

Despite various challenges (technical complexity, extended treatment duration, and higher costs), compared with autotransplantation, orthodontic mesialization achieves vital pulp preservation. This strategy must be implemented by specialized orthodontists.

## References

[1] Ye, Z. X., Qian, W. H., Wu, Y. B. & Yang, C. Pathologies associated with the mandibular third molar impaction. *Sci. Prog.* **104**, 368504211013247 (2021). 33913399 10.1177/00368504211013247 PMC10454952

[2] Chisci, D., Parrini, S., Baldini, N. & Chisci, G. Patterns of third-molar-pericoronitis-related pain: a morphometrical observational retrospective study. *Healthcare* **11**, 1890 (2023). 37444724 10.3390/healthcare11131890 PMC10340319

[3] Saputri, R. I. et al. Is third molar development affected by third molar impaction or impaction-related parameters?. *Clin. Oral. Investig.* **25**, 6681–6693 (2021). 33934201 10.1007/s00784-021-03955-z

[4] Ren, J. G. & Zhao, J. H. Preliminary discussion on the whole life-cycle management of wisdom teeth health. *Zhonghua Kou Qiang Yi Xue Za Zhi* **59**, 760–765 (2024). 39036905 10.3760/cma.j.cn112144-20240426-00165

[5] AlQahtani, S. J., Hector, M. P. & Liversidge, H. M. Brief communication: the London atlas of human tooth development and eruption. *Am. J. Phys. Anthropol.* **142**, 481–490 (2010). 20310064 10.1002/ajpa.21258

[6] Quek, S. L., Tay, C. K., Tay, K. H., Toh, S. L. & Lim, K. C. Pattern of third molar impaction in a Singapore Chinese population: a retrospective radiographic survey. *Int. J. Oral. Maxillofac. Surg.* **32**, 548–552 (2003). 14759117

[7] Banu, A. M. et al. Craniofacial morphology and its relation to the eruption pattern of permanent teeth in the supporting zone of the dentition in a group of Romanian children in Timisoara. *Rom. J. Morphol. Embryol.* **59**, 491–497 (2018). 30173253

[8] Chu, F. C. et al. Prevalence of impacted teeth and associated pathologies-a radiographic study of the Hong Kong Chinese population. *Hong. Kong Med. J.* **9**, 158–163 (2003). 12777649

[9] Li, F. L., Meng, J. R., Guo, Y., Feng, F. W. & Sun, Y. G. The epidemiological features of 1 777 patients with impacted mandibular third molar and their effect on drysocket morbidity after tooth extraction. *J. Pract. Stomatol.* **35**, 818–821 (2019).

[10] Li, L. L. & Xiang, G. L. Cone-beam CT study on growth and development of mandibular third molars among 562 adolescents aged 12-16 in Wuhan. *J. Oral. Sci. Res.* **39**, 217–220 (2023).

[11] Jacques, E., Ebogo, M., Eng, Y. C., Donald, N. & Odile, Z. Radiographic evaluation of impacted third mandibular molar according to the classification of winter, Pell and Gregory in a sample of Cameroonian population. *Ethiop. J. Health Sci.* **33**, 851–858 (2023). 38784512 10.4314/ejhs.v33i5.15 PMC11111199

[12] Faadiya, A. N., Widyaningrum, R., Arindra, P. K. & Diba, S. F. The diagnostic performance of impacted third molars in the mandible: a review of deep learning on panoramic radiographs. *Saudi Dent. J.* **36**, 404–412 (2024). 38525176 10.1016/j.sdentj.2023.11.025 PMC10960107

[13] Sun, R., Sun, Y. Q., Cai, Y. & Zhao, J. Palatal approach for surgical removal of mesioangularly impacted maxillary third molar - a pilot study. *BMC Oral. Health* **23**, 518 (2023). 37491236 10.1186/s12903-023-03234-1 PMC10369702

[14] Nguyen, B. T., Nguyen-Le, C. T., Nguyen, B. T. & Le, S. H. Multivariable analysis of correlation between anatomical features of mandibular third molars and pericoronitis. *Int. J. Dent.* **2024**, 8260559 (2024). 39703785 10.1155/ijod/8260559 PMC11658846

[15] Kim, J. Y. et al. Are there differences in the causes and complications of mandibular third molar extraction in older patients compared to younger patients?. *J. Oral. Maxillofac. Surg.* **82**, 1416–1424 (2024). 39038596 10.1016/j.joms.2024.06.182

[16] Zhou, W., Xiong, Z., Fan, J., Yang, T. & Gu, Y. Cone-beam computed tomographic investigation of the association between impacted mandibular third molars and the development of distal caries in adjacent second molars in a Chinese population. *Heliyon* **10**, e40655 (2024). 39654787 10.1016/j.heliyon.2024.e40655 PMC11626789

[17] Wang, D. et al. External root resorption of the second molar associated with mesially and horizontally impacted mandibular third molar: evidence from cone beam computed tomography. *Clin. Oral. Investig.* **21**, 1335–1342 (2017). 27316639 10.1007/s00784-016-1888-y

[18] Gao, R. et al. Presence of nonimpacted third molars affect the response of neighboring teeth to nonsurgical periodontal therapy. *J. Periodontol*. 10.1002/JPER.24-0674 (2025). 10.1002/JPER.24-0674 39925314

[19] Brignardello-Petersen, R. The prevalence of odontogenic cysts and tumors associated with impacted third molars is probably close to 5. *J. Am. Dent. Assoc.* **150**, e161 (2019). 31375211 10.1016/j.adaj.2019.05.015

[20] Cardoso, G. B., Savegnago, G. D. O., Hirsch, W. D. B., Vizzotto, M. B. & Liedke, G. S. Pathologic conditions associated with impacted third molars: a retrospective study of panoramic radiographs in a Southern Brazilian population. *Imaging Sci. Dent.* **53**, 303–312 (2023). 38174038 10.5624/isd.20230036 PMC10761288

[21] Durham, J. Oral surgery: part 3. Temporomandibular disorders. *Br. Dent. J.* **215**, 331–337 (2013). 24113949 10.1038/sj.bdj.2013.950

[22] Friesen, R., Li, X., Singh, V. & Pacheco-Pereira, C. Temporomandibular joint disorders and pain confounders: an awareness study. *Int. Dent. J.* **75**, 824–831 (2025). 39107151 10.1016/j.identj.2024.07.013 PMC11976556

[23] Hounsome, J. et al. Prophylactic removal of impacted mandibular third molars: a systematic review and economic evaluation. *Health Technol. Assess.* **24**, 1–116 (2020). 32589125 10.3310/hta24300 PMC7336222

[24] Chisci, G. et al. The management of a geriatric patient using dabigatran therapy on dentigerous cyst with oral bleeding. *J. Clin. Med.* **13**, 1499 (2024). 38592423 10.3390/jcm13051499 PMC10934523

[25] Wei, X. Z. et al. Effect of preemptive analgesia with ibuprofen on postoperative pain after mandibular third molar extraction: a randomized controlled trial. *Zhonghua Kou Qiang Yi Xue Za Zhi* **59**, 230–236 (2024). 38432654 10.3760/cma.j.cn112144-20231203-00276

[26] Zhou, J. & Zhang, W. Clinical research and application of preemptive analgesia in dental treatment. *Zhonghua Kou Qiang Yi Xue Za Zhi* **57**, 490–494 (2022). 35484671 10.3760/cma.j.cn112144-20220308-00096

[27] Kumar, K. et al. Efficacy of 2% lignocaine and 4% articaine in oral surgical procedure: a comparative study. *J. Contemp. Dent. Pract.* **21**, 1146–1149 (2020). 33686037

[28] Yang, F. et al. Local anaesthesia for surgical extraction of mandibular third molars: a systematic review and network meta-analysis. *Clin. Oral. Investig.* **24**, 3781–3800 (2020). 32833132 10.1007/s00784-020-03490-3

[29] Staedt, H. et al. Buffered 4% articaine reduces pain and enhances anesthesia in maxillary third molar extractions: a randomized, double-blind split-mouth study. *Biomedicines* **12**, 2691 (2024). 39767598 10.3390/biomedicines12122691 PMC11673001

[30] Santos-Sanz, L., Toledano-Serrabona, J. & Gay-Escoda, C. Safety and efficacy of 4% articaine in mandibular third-molar extraction: a systematic review and meta-analysis of randomized clinical trials. *J. Am. Dent. Assoc.* **151**, 912–923.e10 (2020). 33228884 10.1016/j.adaj.2020.08.016

[31] Zhang, G. et al. Improved sedation for dental extraction by using video eyewear in conjunction with nitrous oxide: a randomized, controlled, cross-over clinical trial. *Oral. Surg. Oral. Med. Oral. Pathol. Oral. Radiol.* **113**, 188–192 (2012). 22677735 10.1016/j.tripleo.2011.02.001

[32] Abdullah, W. A., Sheta, S. A. & Nooh, N. S. Inhaled methoxyflurane (Penthrox) sedation for third molar extraction: a comparison to nitrous oxide sedation. *Aust. Dent. J.* **56**, 296–301 (2011). 21884146 10.1111/j.1834-7819.2011.01350.x

[33] Zhang, H. Y., Su, L. W., Sun, H. & Wu, Y. A comparative study of the intraoperative and postoperative response of electric dental handpieces and turbo-pneumatic dental handpieces after mandibular third molar extraction. *China J. Oral. Maxillofac. Surg.* **22**, 440–445 (2024).

[34] Parrini, S., Bovicelli, A. & Chisci, G. Microbiological retention on PTFE versus silk suture: a quantitative pilot study in third molar surgery. *Antibiotics* **12**, 562 (2023). 36978429 10.3390/antibiotics12030562 PMC10044079

[35] Demirjian, A., Goldstein, H. & Tanner, J. M. A new system of dental age assessment. *Hum. Biol.* **45**, 211–227 (1973). 4714564

[36] Yoshida, Y., Shingu, T., Harada, Y., Ida, S. & Takubo, K. A case of pediatric Garre’s osteomyelitis caused by germ infection in the lower impacted wisdom tooth. *Yonago Acta Med.* **66**, 292–296 (2023). 37229369 10.33160/yam.2023.05.005 PMC10203645

[37] Zhang, J. et al. Full life cycle changes of low impacted mandibular third molar associated cystic lesions and adjacent tooth root resorption: a retrospective study. *BMC Oral. Health* **24**, 515 (2024). 38698359 10.1186/s12903-024-04248-z PMC11064400

[38] Esan, T. & Schepartz, L. A. Third molar impaction and agenesis: influence on anterior crowding. *Ann. Hum. Biol.* **44**, 46–52 (2017). 26856343 10.3109/03014460.2016.1151549

[39] Zheng, L. W., Sun, R., Liu, Y. R. X., Lin, L. Z. & Zhao, J. H. Effect of mandibular third molar tooth germ extraction on mandibular development: a retrospective study. *Zhonghua Kou Qiang Yi Xue Za Zhi* **59**, 799–804 (2024). 39036911 10.3760/cma.j.cn112144-20240426-00166

[40] Ghaeminia, H. et al. Surgical removal versus retention for the management of asymptomatic disease-free impacted wisdom teeth. *Cochrane Database Syst. Rev.* **5**, CD003879 (2020). 32368796 10.1002/14651858.CD003879.pub5 PMC7199383

[41] Brunello, G., De Biagi, M., Crepaldi, G., Rodrigues, F. I. & Sivolella, S. An observational cohort study on delayed-onset infections after mandibular third-molar extractions. *Int. J. Dent.* **2017**, 1435348 (2017). 28607555 10.1155/2017/1435348 PMC5457748

[42] Sun, R., Cai, Y., Yuan, Y. & Zhao, J. H. The characteristics of adjacent anatomy of mandibular third molar germs: a CBCT study to assess the risk of extraction. *Sci. Rep.* **7**, 14154 (2017). 29074859 10.1038/s41598-017-14144-y PMC5658424

[43] Yadav, S., Verma, A. & Sachdeva, A. Assessment of lingual nerve injury using different surgical variables for mandibular third molar surgery: a clinical study. *Int. J. Oral. Maxillofac. Surg.* **43**, 889–893 (2014). 24582384 10.1016/j.ijom.2014.01.013

[44] Haug, R. H., Abdul-Majid, J., Blakey, G. H. & White, R. P. Evidenced-based decision making: the third molar. *Dent. Clin. North Am.* **53**, 77–96 (2009). 19215746 10.1016/j.cden.2008.09.004

[45] Wang, B. et al. Does the “root removal first” strategy prevent postoperative complications in the surgical removal of impacted mandibular third molars in the Pell and Gregory class C and horizontal position? - a randomized clinical trial. *BMC Oral. Health* **23**, 391 (2023). 37316782 10.1186/s12903-023-03086-9 PMC10268342

[46] Sun, R. et al. Is it safe and effective to extract impacted maxillary tooth adjacent to maxillary sinus via submaxillary sinus membrane space approach?-a randomized controlled trial. *Clin. Oral. Investig.* **27**, 6081–6087 (2023). 37624523 10.1007/s00784-023-05223-8

[47] Ma, Z. G. et al. An orthodontic technique for minimally invasive extraction of impacted lower third molar. *J. Oral. Maxillofac. Surg.* **71**, 1309–1317 (2013). 23763903 10.1016/j.joms.2013.03.025

[48] Knutsson, K., Lysell, L. & Rohlin, M. Postoperative status after partial removal of the mandibular third molar. *Swed. Dent. J.* **13**, 15–22 (1989). 2734696

[49] Zhang, H. X., Yan, Z. Y., Cui, N. H., Sun, F. & Wu, B. Z. Accuracy of computer-assisted dynamic navigation when performing coronectomy of the mandibular third molar: a pilot study. *J. Dent.* **139**, 104762 (2023). 37898432 10.1016/j.jdent.2023.104762

[50] Yan, Z. Y. et al. Computer-aided three-dimensional assessment of periodontal healing distal to the mandibular second molar after coronectomy of the mandibular third molar: a prospective study. *BMC Oral. Health* **20**, 264 (2020). 32972396 10.1186/s12903-020-01250-z PMC7513308

[51] Yan, Z. Y. et al. Somatosensory changes in Chinese patients after coronectomy vs. total extraction of mandibular third molar: a prospective study. *Clin. Oral. Investig.* **24**, 3017–3028 (2020). 31853899 10.1007/s00784-019-03169-4

[52] Yan, Z. Y. et al. Three-dimensional assessment of root migration and rotation patterns after coronectomy: bone-embedded roots versus soft tissue-covered roots. *Int. J. Oral. Maxillofac. Surg.* **50**, 699–706 (2021). 33069515 10.1016/j.ijom.2020.09.015

[53] Wang, F. et al. A comparative study of a two-stage surgical approach combining coronectomy with microimplant anchorage traction for extraction of impacted mandibular third molars with different traction angles. *Zhonghua Kou Qiang Yi Xue Za Zhi* **59**, 792–798 (2024). 39036910 10.3760/cma.j.cn112144-20240507-00184

[54] Pang, S. L. et al. Third molar coronectomy vs total removal in second molar periodontal healing. *Int. Dent. J.* **74**, 246–252 (2024). 37666687 10.1016/j.identj.2023.08.006 PMC10988259

[55] Peixoto, A. O. et al. Benefits of coronectomy in lower third molar surgery: a systematic review and meta-analysis. *J. Oral. Maxillofac. Surg.* **82**, 73–92 (2024). 37925166 10.1016/j.joms.2023.09.024

[56] He, L. et al. Digital technology-assisted extraction of impacted maxillary third molar located between the furcation of maxillary second molar by root dislocation: a case report. *Hua Xi Kou Qiang Yi Xue Za Zhi* **42**, 403–408 (2024). 39049662 10.7518/hxkq.2024.2023362 PMC11190866

[57] Zeng, J. et al. Research on a novel digital tooth sectioning guide system for tooth sectioning during mandibular third molar extraction: an in vitro study. *J. Stomatol. Oral. Maxillofac. Surg.* **124**, 101383 (2023). 36646285 10.1016/j.jormas.2023.101383

[58] Han, L. Z. et al. Digital robot-assisted minimally invasive impacted tooth extraction: a case report. *Heliyon* **10**, e36787 (2024). 39286173 10.1016/j.heliyon.2024.e36787 PMC11402922

[59] Wang, Y. et al. Digital design combined with endoscopic minimally invasive extraction of impacted mandibular third molars with roots in contact with the mandibular canal. *Zhonghua Kou Qiang Yi Xue Za Zhi* **59**, 1221–1227 (2024). 39606980 10.3760/cma.j.cn112144-20240512-00201

[60] Jiang, J., Chen, K., Wang, E., Duan, D. & Xu, X. Endoscopically-assisted extraction of broken roots or fragments within the mandibular canal: a retrospective case series study. *BMC Oral. Health* **24**, 456 (2024). 38622566 10.1186/s12903-024-04216-7 PMC11020977

[61] Jiang, J. Q. et al. Endoscopic visualization of the inferior alveolar nerve associated with somatosensory changes after impacted mandibular third molar extraction. *Odontology* **111**, 982–992 (2023). 36773195 10.1007/s10266-023-00788-y PMC10492667

[62] Ku, J. K. & Jeong, Y. K. Effectiveness of bone graft for an alveolar defect on adjacent second molar after impacted mandibular third molar extraction. *J. Oral. Maxillofac. Surg.* **79**, 756–762 (2021). 33359105 10.1016/j.joms.2020.11.030

[63] Rodrigues, E. D., Pontual, A. D., Macedo, R. A., Nascimento, E. & Vasconcelos, B. C. Evaluation of bone repair with platelet-rich fibrin following the extraction of impacted third molars - randomized clinical trial. *Med. Oral. Patol. Oral. Cir. Bucal* **28**, e433–e441 (2023). 37330965 10.4317/medoral.25856 PMC10499344

[64] Thuruthel, M. J., Kumar, L. K. S., Kurien, N. M. & Tharakan, M. Efficacy of gelatamp in controlling the postoperative sequelae following mandibular posterior teeth extraction - a split-mouth study. *J. Oral. Biol. Craniofac. Res.* **13**, 96–103 (2023). 36561420 10.1016/j.jobcr.2022.11.006 PMC9763509

[65] Fan, G., Shen, Y., Cai, Y., Zhao, J. H. & Wu, Y. Uncontrollable bleeding after tooth extraction from asymptomatic mild hemophilia patients: two case reports. *BMC Oral. Health* **22**, 69 (2022). 35282827 10.1186/s12903-022-02074-9 PMC8919556

[66] Oladega, A. A., James, O. & Adeyemo, W. L. Cyanoacrylate tissue adhesive or silk suture for closure of surgical wound following removal of an impacted mandibular third molar: a randomized controlled study. *J. Craniomaxillofac. Surg.* **47**, 93–98 (2019). 30501926 10.1016/j.jcms.2018.10.018

[67] Parrini, S., Arzente, G., Bartali, E. & Chisci, G. The role of cyanoacrylate after mandibular third molar surgery: a single center study. *Bioengineering* **11**, 569 (2024). 38927805 10.3390/bioengineering11060569 PMC11200889

[68] Low, S. H., Lu, S. L. & Lu, H. K. Evidence-based clinical decision making for the management of patients with periodontal osseous defect after impacted third molar extraction: a systematic review and meta-analysis. *J. Dent. Sci.* **16**, 71–84 (2021). 33384781 10.1016/j.jds.2020.06.018 PMC7770311

[69] Siawasch, S. A. M. et al. Autologous platelet concentrates after third molar extraction: a systematic review. *Periodontol 2000* **97**, 131–152 (2025). 39318055 10.1111/prd.12600

[70] Park, H. C. et al. Early bone formation at a femur defect using CGF and PRF grafts in adult dogs: a comparative study. *Implant Dent.* **25**, 387–393 (2016). 27123893 10.1097/ID.0000000000000423

[71] Bonazza, V. et al. Growth factors release from concentrated growth factors: effect of beta-tricalcium phosphate addition. *J. Craniofac. Surg.* **29**, 2291–2295 (2018). 29771832 10.1097/SCS.0000000000004607

[72] Zhang, H., Dong, J., Wang, X., Sun, X. & Wang, J. Application of concentrated growth factor in mandibular third molar extraction: a protocol for systematic review and meta-analysis. *PLoS One* **19**, e0302581 (2024). 38696507 10.1371/journal.pone.0302581 PMC11065272

[73] Elayah, S. A. et al. Effect of concentrated growth factor (CGF) on postoperative sequel of completely impacted lower third molar extraction: a randomized controlled clinical study. *BMC Oral. Health* **22**, 368 (2022). 36042448 10.1186/s12903-022-02408-7 PMC9426240

[74] Sun, S. et al. A novel concentrated growth factor (CGF) and bio-oss based strategy for second molar protection after impacted mandibular third molar extraction: a randomized controlled clinical study. *BMC Oral. Health* **23**, 750 (2023). 37828455 10.1186/s12903-023-03411-2 PMC10571244

[75] Ozzo, S. & Kheirallah, M. The efficiency of two different synthetic bone graft materials on alveolar ridge preservation after tooth extraction: a split-mouth study. *BMC Oral. Health* **24**, 1040 (2024). 39232718 10.1186/s12903-024-04803-8 PMC11375842

[76] Campana, V. et al. Bone substitutes in orthopaedic surgery: from basic science to clinical practice. *J. Mater. Sci. Mater. Med.* **25**, 2445–2461 (2014). 24865980 10.1007/s10856-014-5240-2 PMC4169585

[77] Barootchi, S. et al. Alveolar ridge preservation: complications and cost-effectiveness. *Periodontol 2000* **92**, 235–262 (2023). 36580417 10.1111/prd.12469

[78] Wushou, A., Zheng, Y., Han, Y., Yang, Z. C. & Han, F. K. The use of autogenous tooth bone graft powder in the treatment of osseous defects after impacted mandibular third molar extraction: a prospective split-mouth clinical pilot study. *BMC Oral. Health* **22**, 433 (2022). 36184595 10.1186/s12903-022-02473-y PMC9526982

[79] Sivolella, S., Brunello, G., Fistarol, F., Stellini, E. & Bacci, C. The bone lid technique in oral surgery: a case series study. *Int. J. Oral. Maxillofac. Surg.* **46**, 1490–1496 (2017). 28716472 10.1016/j.ijom.2017.06.027

[80] Sivolella, S. et al. The bone lid technique in oral and maxillofacial surgery: a scoping review. *J. Clin. Med.* **11**, 3667 (2022). 35806950 10.3390/jcm11133667 PMC9267370

[81] Susarla, S. M., Blaeser, B. F. & Magalnick, D. Third molar surgery and associated complications. *Oral. Maxillofac. Surg. Clin. North Am.* **15**, 177–186 (2003). 18088673 10.1016/S1042-3699(02)00102-4

[82] Xu, G. Z. et al. Anatomic relationship between impacted third mandibular molar and the mandibular canal as the risk factor of inferior alveolar nerve injury. *Br. J. Oral. Maxillofac. Surg.* **51**, e215–e219 (2013). 23411471 10.1016/j.bjoms.2013.01.011

[83] Sarikov, R. & Juodzbalys, G. Inferior alveolar nerve injury after mandibular third molar extraction: a literature review. *J. Oral. Maxillofac. Res.* **5**, e1 (2014). 25635208 10.5037/jomr.2014.5401 PMC4306319

[84] Hillerup, S. Iatrogenic injury to oral branches of the trigeminal nerve: records of 449 cases. *Clin. Oral. Investig.* **11**, 133–142 (2007). 17186310 10.1007/s00784-006-0089-5

[85] Chai, Y. et al. Risk factors associated with inferior alveolar nerve injury after extraction of impacted lower mandibular third molars: a prospective cohort study. *J. Oral. Maxillofac. Surg.* **82**, 1100–1108 (2024). 38821486 10.1016/j.joms.2024.05.003

[86] Lu, Z., Bingquan, H., Jun, T. & Fei, G. Effectiveness of concentrated growth factor and laser therapy on wound healing, inferior alveolar nerve injury and periodontal bone defects post-mandibular impacted wisdom tooth extraction: a randomized clinical trial. *Int. Wound J.* **21**, e14651 (2024). 38272792 10.1111/iwj.14651 PMC10789919

[87] Huang, Z. Q. et al. Removal of the residual roots of mandibular wisdom teeth in the lingual space of the mandible via endoscopy. *Int. J. Oral. Maxillofac. Surg.* **44**, 400–403 (2015). 25543902 10.1016/j.ijom.2014.11.018

[88] Hu, Y. K., Yang, C., Zhou Xu, G., Wang, Y. & Abdelrehem, A. Retrieval of root fragment in maxillary sinus via anterolateral wall of the sinus to preserve alveolar bone. *J. Craniofac. Surg.* **26**, e81–e84 (2015). 25723657 10.1097/SCS.0000000000001286

[89] Sun, Y. Q., Sun, R. & Zhao, J. H. The efficacy of minocycline hydrochloride ointment versus iodoform gauze for alveolar osteitis: a prospective cohort study. *BMC Oral. Health* **22**, 448 (2022). 36258229 10.1186/s12903-022-02468-9 PMC9580180

[90] Torul, D., Omezli, M. M. & Avci, T. Investigation of the clinical efficacy of CGF and ozone in the management of alveolar osteitis: a randomized controlled trial. *Clin. Oral. Investig.* **27**, 4521–4529 (2023). 37231273 10.1007/s00784-023-05074-3

[91] Giansiracusa, A., Parrini, S., Baldini, N., Bartali, E. & Chisci, G. The effect of photobiomodulation on third molar wound recovery: a systematic review with meta-analysis. *J. Clin. Med.* **13**, 5402 (2024). 39336889 10.3390/jcm13185402 PMC11432466

[92] Liao, F., Wang, H., Zhao, J., Zhang, B. & Zhong, H. Effectiveness evaluation of autotransplanted teeth after performing extraoral endodontic surgery instead of conventional root canal therapy. *BMC Oral. Health* **23**, 1005 (2023). 38097962 10.1186/s12903-023-03733-1 PMC10722803

[93] Huang, J. et al. Outcomes of autotransplanted third molars with complete root formation: a systemic review and meta-analysis. *J. Evid. Based Dent. Pract.* **23**, 101842 (2023). 37201977 10.1016/j.jebdp.2023.101842

[94] Chung, W. C., Tu, Y. K., Lin, Y. H. & Lu, H. K. Outcomes of autotransplanted teeth with complete root formation: a systematic review and meta-analysis. *J. Clin. Periodontol.* **41**, 412–423 (2014). 24393101 10.1111/jcpe.12228

[95] Plotino, G. et al. European Society of Endodontology position statement: surgical Extrusion, intentional replantation and tooth autotransplantation: European Society of Endodontology developed by. *Int. Endod. J.* **54**, 655–659 (2021). 33501680 10.1111/iej.13456

[96] Professional Committee of Teeth and Alveolar Surgery & Chinese Stomatological Association Chinese expert consensus on standardized operation process of autotransplantation of teeth. China J. Oral. Maxillofac. Surg. 18, 390–394 (2020)..

[97] Tsukiboshi, M., Tsukiboshi, C. & Levin, L. A step-by step guide for autotransplantation of teeth. *Dent. Traumatol.* **39**, 70–80 (2023). 36655600 10.1111/edt.12819

[98] Lucas-Taule, E. et al. Mid-term outcomes and periodontal prognostic factors of autotransplanted third molars: a retrospective cohort study. *J. Periodontol.* **92**, 1776–1787 (2021). 33764523 10.1002/JPER.21-0074

[99] Yang, S., Jung, B. Y. & Pang, N. S. Outcomes of autotransplanted teeth and prognostic factors: a 10-year retrospective study. *Clin. Oral. Investig.* **23**, 87–98 (2019). 29525925 10.1007/s00784-018-2412-3

[100] Yu, H. J., Jia, P., Lv, Z. & Qiu, L. X. Autotransplantation of third molars with completely formed roots into surgically created sockets and fresh extraction sockets: a 10-year comparative study. *Int. J. Oral. Maxillofac. Surg.* **46**, 531–538 (2017). 28062250 10.1016/j.ijom.2016.12.007

[101] Wu, Y. et al. Autotransplantation of mature impacted tooth to a fresh molar socket using a 3D replica and guided bone regeneration: two years retrospective case series. *BMC Oral. Health* **19**, 248 (2019). 31727038 10.1186/s12903-019-0945-8 PMC6857220

[102] Almpani, K., Papageorgiou, S. N. & Papadopoulos, M. A. Autotransplantation of teeth in humans: a systematic review and meta-analysis. *Clin. Oral. Investig.* **19**, 1157–1179 (2015). 25903060 10.1007/s00784-015-1473-9

[103] Jang, Y. et al. Prognostic factors for clinical outcomes in autotransplantation of teeth with complete root formation: survival analysis for up to 12 years. *J. Endod.* **42**, 198–205 (2016). 26686824 10.1016/j.joen.2015.10.021

[104] Kahler, B., Hu, J. Y., Marriot-Smith, C. S. & Heithersay, G. S. Splinting of teeth following trauma: a review and a new splinting recommendation. *Aust. Dent. J.* **61**, 59–73 (2016). 26923448 10.1111/adj.12398

[105] Lin, P. Y., Chiang, Y. C., Hsu, L. Y., Chang, H. J. & Chi, L. Y. Endodontic considerations of survival rate for autotransplanted third molars: a nationwide population-based study. *Int. Endod. J.* **53**, 733–741 (2020). 32009248 10.1111/iej.13273

[106] Baik, U. B., Chun, Y. S., Jung, M. H. & Sugawara, J. Protraction of mandibular second and third molars into missing first molar spaces for a patient with an anterior open bite and anterior spacing. *Am. J. Orthod. Dentofac. Orthop.* **141**, 783–795 (2012). 10.1016/j.ajodo.2010.07.031 22640680

[107] Baik, U. B., Kim, M. R., Yoon, K. H., Kook, Y. A. & Park, J. H. Orthodontic uprighting of a horizontally impacted third molar and protraction of mandibular second and third molars into the missing first molar space for a patient with posterior crossbites. *Am. J. Orthod. Dentofac. Orthop.* **151**, 572–582 (2017). 10.1016/j.ajodo.2016.01.019 28257742

[108] Lee, M. Y., Park, J. H., Pang, K. M., Chang, N. Y. & Chae, J. M. Interdisciplinary treatment of a mutilated dentition with multiple missing teeth and horizontal impaction of a mandibular third molar using an orthodontic skeletal anchorage and dental implants-Case report. *J. Esthet. Restor. Dent.* **36**, 1502–1510 (2024). 39012042 10.1111/jerd.13281

